# Cardiac alterations following experimental hip fracture - inflammaging as independent risk factor

**DOI:** 10.3389/fimmu.2022.895888

**Published:** 2022-09-05

**Authors:** Ina Lackner, Birte Weber, Jochen Pressmar, Anna Odwarka, Charles Lam, Melanie Haffner-Luntzer, Ralph Marcucio, Theodore Miclau, Miriam Kalbitz

**Affiliations:** ^1^ Department of Trauma and Orthopedic Surgery, University Hospital Erlangen, Friedrich-Alexander University Erlangen-Nuremberg, Erlangen, Germany; ^2^ Department of Traumatology, Hand, Plastic, and Reconstructive Surgery, University Medical Center Ulm, Ulm, Germany; ^3^ Department of Orthopaedic Surgery, Orthopaedic Trauma Institute, Zuckerberg San Francisco General Hospital, University of California, San Francisco, San Francisco, CA, United States; ^4^ Department of Trauma, Hand and Reconstructive Surgery, Goethe University of Frankfurt, Frankfurt, Germany; ^5^ Institute of Orthopaedic Research and Biomechanics, University Medical Center Ulm, Ulm, Germany

**Keywords:** proximal femur fracture, hip fracture, secondary cardiac injury, cardiac inflammation, cardiac structure, inflammaging

## Abstract

**Background:**

Cardiac injuries following trauma are associated with a worse clinical outcome. So-called trauma-induced secondary cardiac injuries have been recently described after experimental long bone fracture even in absence of direct heart damage. With the progressive aging of our society, the number of elderly trauma victims rises and therefore the incidence of hip fractures increases. Hip fractures were previously shown to be associated with adverse cardiac events in elderly individuals, which have mainly been attributed to pre-conditioned cardiac diseases. The aim of the present study was to investigate the effect of hip fractures on the heart in healthy young and middle-aged mice.

**Materials and Methods:**

Young (12-week-old) and middle-aged (52-week-old) female C57BL/6 mice either received an intramedullary stabilized proximal femur fracture or sham treatment. The observation time points included 6 and 24 h. Systemic levels of pro-inflammatory mediators as well as local inflammation and alterations in myocardial structure, metabolism and calcium homeostasis in left ventricular tissue was analyzed following hip fracture by multiplex analysis, RT-qPCR and immunohistochemistry.

**Results:**

After hip fracture young and middle-aged mice showed increased systemic IL-6 and KC levels, which were significantly elevated in the middle-aged animals. Furthermore, the middle-aged mice showed enhanced myocardial expression of HMGB1, TLR2/4, TNF, IL1β and NLRP3 as well as considerable alterations in the myocardial expression of glucose- and fatty acid transporters (HFABP, GLUT4), calcium homeostasis proteins (SERCA) and cardiac structure proteins (desmin, troponin I) compared to the young animals following hip fracture.

**Conclusion:**

Young and middle-aged mice showed local myocardial alterations, which might predispose for the development of secondary cardiac injury following hip fracture. Age and the age-associated phenomenon of ‘inflammaging’ seemed to be an independent risk factor aggravating and accelerating cardiac alterations following hip fracture.

## Introduction

Worldwide, trauma is the leading cause of death and disability within the young population (1, 2). Blunt cardiac injuries following severe trauma were shown to be an independent predictor for a worse clinical outcome and where primarily linked to blunt mechanical myocardial damage (3, 4). Moreover, so-called trauma-induced secondary cardiac injuries (TISCI) have also been described even in absence of direct mechanical heart damage, correlating with long-term morbidity and mortality of the patients (5–7). Trauma-induced secondary cardiac injuries imply the clinical occurrence of adverse cardiac events such as acute coronary syndrome, atrial fibrillation, myocardial depression and ventricular arrhythmia in severely injured patients without mechanical heart damage and are characterized by elevated systemic levels of troponin I and heart fatty acid binding protein (HFABP) (5–7). The development of these secondary cardiac injuries is mostly linked to an exuberant systemic inflammatory response following trauma (5–7).

Recently, our group described secondary cardiac injuries after experimental long bone fracture (8–10). In these studies, pigs showed an impaired cardiac function and valvular insufficiencies 6 h following femur fracture, which was primarily linked to an increased systemic but also local cardiac inflammation, all of which was further observed in mice with isolated diaphyseal femur or tibia fracture (8–10). Cardiac inflammation following bone fracture was mediated *via* damage-associated molecular patterns (DAMPs), toll-like receptor (TLR) signaling as well as by the activation of the complement system and the NLR-pyrin domain containing protein 3 (NLRP3) inflammasome (8–10). Besides inflammation, alterations in cardiac structure, metabolism and calcium handling proteins were considered to contribute to the development of secondary cardiac injuries following long bone fracture (8–10).

With the progressive aging of our society, the number of elderly trauma victims rises correspondingly (11). As a consequence, the incidence of hip fractures increases rapidly (12) and the number of these fractures is expected to reach 4.5 million by the year 2050 (13). Hip fractures primarily occur in elderly individuals aged 70-80 years with low bone mass and are associated with a long-term morbidity and increased mortality. Moreover, distinct age-related co-morbidities were shown to be independent risk factors in elderly hip fracture patients, increasing their in-hospital mortality 3-fold following hip fracture (14). Amongst others, these age-related co-morbidities included several cardiovascular diseases and their development was shown to accelerate with increased aging (15). With respect to secondary cardiac injury, a clinical study showed electrocardiographic abnormalities in patients with traumatic hip fractures, such as atrial fibrillation, abnormal QTc prolongation, sinus tachycardia and sinus bradycardia compared to patients with non-traumatic hip surgery (16). Another clinical study demonstrated the development of major cardiac events (all-cause deaths, heart failure, new-onset atrial fibrillation, myocardial infarction, and cardiovascular re-hospitalization) in elderly patients within 90 days following hip fracture (17). In this study, the patients’ age correlated with systemic levels of troponin and brain natriuretic peptide, with reduced ejection fraction and with other major adverse cardiac events (17). In earlier clinical studies, the occurrence of coronary heart disease was shown to be two times more frequent in patients with hip fracture compared to healthy controls (18, 19). However, in all of the above-mentioned studies, the majority of patients suffered from pre-conditioned cardiac diseases such as myocardial infarction and congestive heart failure (16). It was further shown that pre-conditioned cardiac diseases and impaired cardiac function such as a 50% reduced left ventricular ejection fraction (LVEF), increase the occurrence of major adverse cardiac events following hip fracture in elderly individuals and were therefore considered as independent risk factors for a worse clinical outcome in these patients (17, 20, 21). Additionally, age >75 years appeared to be another independent risk factor for an increased mortality (20, 22) but also for the occurrence of major adverse cardiac events following hip fracture (17).

Additionally, several clinical studies showed an increased systemic inflammatory response following hip fracture in elderly individuals, which was also associated with an increased mortality rate (23). Other studies demonstrated enhanced susceptibility to infections post hip fracture surgery, which was linked to a dysregulated immune response in the elderly patients (24), correlating also with an increased mortality (25).

In conclusion, these clinical studies showed that age is an independent risk factor for an increased mortality following hip fracture in the elderly and that pre-conditioned cardiac diseases aggravate this pathologic condition. With respect to secondary cardiac injury, the occurrence of major cardiac events following hip fracture was mostly linked to pre-conditioned cardiac diseases in combination with an advanced age of the patients. So far, it is unknown whether hip fractures induce myocardial damage in younger individuals, resulting in the pathologic condition of secondary cardiac injuries. Moreover, it remains to be clarified whether age is an independent risk factor aggravating secondary cardiac injuries following hip fracture in absence of pre-conditioned cardiac diseases.

Therefore, we investigated in the present study whether hip fractures induce cardiac inflammation and cardiac alterations in young female mice and whether advanced age is an independent risk factor aggravating these pathologic conditions.

## Materials and methods

### Animals and experimental design

The animal experiments were conducted in collaboration with the University of California (UCSF), San Francisco Orthopaedic Trauma Institute, San Francisco, California, USA. All animal experiments were approved by the local animal welfare committee (IACUC UCSF AN143402-03B) and were performed in accordance with the international regulations for laboratory animal welfare and handling (ARRIVE guidelines). For the present study, 48 female C57BL/6J mice (The Jackson Laboratory) were used in total. The mice were further divided into two age groups. Twenty-four mice with an age between 10-12 weeks (young) and 24 mice with an age between 52-54 (middle-aged) weeks were used for the experiments. We defined the age of the mice according to the guideline by Jackson Laboratories (https://www.jax.org/research-and-faculty/research-labs/the-harrison-lab/gerontology/life-span-as-a-biomarker). Therefore, 3 months old mice were considered as young mice and 12 months old mice were considered as middle-aged mice (see also Flurkey, K; Currer, J M.; and Harrison, D E., “Mouse models in aging research.” (2007). *Faculty Research 2000 - 2009*. 1685.)

Twelve mice of each age group (n=12 young, n=12 middle-aged) received an experimental proximal femur fracture, stabilized by an intramedullary nail, as described previously (26). Control animals of each age group (n=12 young, n=12 middle-aged) underwent sham procedure, including analgesia and anesthesia but without surgical procedure. The animals of each age and treatment group were further randomized into two observation periods of 6 and 24 h, with n=6 animals in each group. Therefore, the following experimental groups were analyzed in the present study: 12-week-old mice after 6 h with proximal femur fracture (n=6) or sham procedure (n=6) and after 24 h with proximal femur fracture (n=6) or sham procedure (n=6). Fifty-two-week-old mice after 6 h with proximal femur fracture (n=6) or sham procedure (n=6) and after 24 h with proximal femur fracture (n=6) or sham procedure (n=6).

### Surgical procedure

The surgical procedure was conducted under general anesthesia, using 50 mg/kg ketamine hydrochloride (Henry Schein Animal Health 100 mg/ml) at a ratio of 1:1 with dexmedetomidine hydrochloride (Orion pharma, 0.5 mg/ml) and analgesia, using 0.05 mg/kg buprenorphine (0.03 mg/ml, injection every 6 hours). The experimental proximal femur fracture was applied as described previously (26). Briefly, a 24G cannula was introduced retrograde into the right femur to stabilize the fracture. Afterwards, a 0.5 cm skin incision was made along the femur. The femoral muscles were separated bluntly. Free access to the proximal femur bone was achieved by cutting the tendon insertion at the third trochanter. With a Gigli wire saw of 0.44 mm diameter, an osteotomy was induced between the third and the lesser trochanters, producing an intertrochanteric proximal femur fracture. Afterwards, the muscles were sutured with a Vicryl 5-0 suture. The skin was closed using a non-absorbable Resolon 5-0 suture. After the experimental procedure, mice were allowed to awake and to move freely directly after surgery. The animals were monitored over the entire observation period. After the respective observation periods of 6 and 24 h, the animals were euthanized by using carbon dioxide.

### Sample collection

After the follow-up period of either 6 ​h or 24 ​h, mice were euthanized using carbon dioxide. Whole blood was taken immediately after euthanasia by cardiac puncture. Plasma samples were collected after centrifugation with 5 ​min (800 × *g*, 4 ​°C) and a second centrifugation step for 2 ​min (13000 × *g*, 4 ​°C). The plasma samples were stored at −80 ​°C until analysis. Samples of left ventricular cardiac tissue were taken immediately after euthanasia and either quick-frozen in liquid nitrogen or fixed in 4% paraformaldehyde for 48 ​h.

### Multiplex analysis

To analyze the systemic inflammation after proximal femur fracture in adult and middle-aged mice, a murine ProcartaPlex Immunoassay (ThermoFisher Scientific, Waltham, MA, USA) was used and systemic levels of interleukin-6 (IL-6), keratinocyte chemoattractant (KC) and interferon gamma (IFNγ) were determined in the plasma of the mice. All procedures were performed according to manufacturer’s instructions.

### Immunohistochemistry (IHC) and immunofluorescence (IF)

For immunohistochemical and immunofluorescence analysis, formalin-fixed and paraffin-embedded tissue sections of the left ventricle were used. Left ventricular tissue sections were dewaxed and rehydrated. Antigen unmasking was performed by boiling the tissue sections in 10 mM citrate buffer (pH 6) at 100°C. Non-specific binding sides were blocked by 10% goat serum. Specific antigen binding was performed by incubating the tissue sections with the respective primary antibodies for C3a receptor (Bioss, Woburn, MA, USA), glutathione peroxidase 4 (GSH) (abcam, Cambridge, UK), High-mobility group box 1 (HMGB1) protein (abcam, Cambridge, UK), desmin (GeneTex, Irvine, CA, USA), α-actinin (GeneTex, Irvine, CA, USA), nitrotyrosine (Merck, Darmstadt, Germany), superoxide dismutase (SOD) (abcam, Cambridge, UK), troponin I (abcam, Cambridge, UK) and tumor necrosis factor (TNF) (abcam, Cambridge, UK) for overnight at 4°C. For immunohistochemical (IHC) staining, a biotin-labelled secondary antibody was used for the detection of specific antibody binding (ThermoFisher, Waltham, MA, USA). Signal amplification was performed by using VECTASTAIN^®^ ABC HRP Kit (Vector Laboratories Inc., Burlingame, CA, USA). Signal development was conducted by using VECTOR^®^ NovaRED™ Peroxidase (HRP) Substrate Kit (Vector Laboratories Inc., Burlingame, CA, USA). Cell nuclei were counterstained with Hematoxylin according to Mayer. For immunofluorescence (IF), an AlexaFluor488^®^-labelled or an AlexaFluor^®^647-labelled secondary antibody were used for the detection of specific antibody binding (Jackson ImmunoResearch Laboratories, Inc., West Grove, PA, USA). Cell nuclei were counterstained with Hoechst33342 (ThermoFisher, Waltham, MA, USA). The sections were investigated by bright field microscopy or by fluorescence microscopy using an Axio Imager M.2 microscope (Zeiss, Jena, Germany). To quantify epitope expression, imaging of three distinct, representative fields of view (40x magnification) were examined for each animal. For quantification of immunohistochemical and immunofluorescent staining, the ZEN 2.3 software (Zeiss, Jena, Germany) was used. For imaging, the ZEN 2.3 software was used. Prior to imaging, the optimal exposure time for the respective antibody staining was determined and standardized for the ZEN 2.3 software, to get an optimal image of each stained section. For analysis of protein expression, a specific threshold for pixel density or fluorescence intensity was manually determined and standardized for each antibody staining (C3aR, HMGB1, TNF, nitrotyrosine, SOD, GSH, desmin, α-actinin, troponin I) prior to the quantification. With the ZEN 2.3 software, each picture was analyzed independently with respect to the defined threshold for pixel density or fluorescence intensity. For each picture a specific mean value of pixel density (IHC) or fluorescence intensity (IF) was calculated by the software. Results are presented as mean pixel density (IHC) or mean fluorescence intensity (IF).

### Hematoxylin and eosin (H.E.) staining

Formalin-fixed and paraffin embedded tissue sections from left ventricles were used. Tissue sections were dewaxed and rehydrated. Myocardial tissue sections were stained with hematoxylin & eosin staining kit (Morphisto, Frankfurt am Main, Germany). For quantification of myocardial damage, a heart injury score was defined as described previously (27, 28). For determination of the heart injury score, the H.E. sections of myocardial tissue were scored for 1) apoptosis, 2) contraction band necrosis, 3) neutrophil infiltration, 4) intramuscular bleeding, 5) rupture, 6) edema and 7) ischemia.

### RNA isolation

RNA was extracted from quick-frozen left ventricular tissue of the mice left ventricle. For this procedure 50 mg of quick-frozen left ventricular tissue was added to 1 ml of Invitrogen TRIzol Reagent (Sigma-Aldrich, St. Louis, MO, USA) and was then homogenized. Left ventricular tissue mRNA was further extracted by using chloroform and purified by using ethanol. mRNA quantity and purity were determined by using Tecan Spark^®^ reader (Tecan Group, Männedorf, Switzerland) and mRNA purity was defined by 260/280 nm ratio.

### Reverse transcribed quantitative polymerase chain reaction (RT-qPCR)

The respective RNA samples were reverse transcribed in cDNA using SuperScript™ IV VILO™ MasterMix with ezDNAse (Invitrogen, Carlsbad, CA, USA). For quantitative PCR, the PowerUp™ SYBR™ Green Master Mix (Applied Biosystems, Waltham, MA, USA) was used. For the reverse cDNA transcription, 200 ng of mRNA were used. Then, 20 ng of cDNA were used for the qPCR. All procedures were performed according to the manufacturer’s instructions. For qPCR the QuantStudio3 system (Applied Biosystems, Waltham, MA, USA) was utilized. Quantitative mRNA expression of murine atrial natriuretic peptide (ANP), brain natriuretic peptide (BNP), C3a receptor (C3aR), C5a receptor 1 (C5aR1), fibroblast growth factor 23 (FGF23), glucose transporter 4 (GLUT4), heart fatty acid binding protein (HFABP), interleukin-1β (IL-1β), toll-like receptor (TLR) 2, TLR4, TLR9, tumor necrosis factor (TNF), NLR family pyrin domain containing 3 (NLRP3), sarcoplasmic/endoplasmic reticulum ATPase (SERCA) and troponin I was examined and calculated by the cycle threshold method ΔΔCt. Respective genes were normalized using housekeeping gene glutaraldehyde-phosphate dehydrogenase (GAPDH). Results are presented as mean fold change. The primer sequences of used primers are listed in **Table 1**.

**Table 1:** Primer sequences.

Gene	Primer sequence
Atrial natriuretic peptide (ANP)	for: 5’-TCCAGGCCATATTGGAGCAA-3’ rev: 5’-GTGGTCTAGCAGGTTCTTGAAAT-3‘
Brain natriuretic peptide (BNP)	for: 5’-AGCTGCTTTGGGCACAAGATA-3‘ rev: 5’-CAACAACTTCAGTGCGTTACAG-3‘
C3a receptor (C3aR)	for: 5‘-CATCGAAACGTGAGAACCGC-3‘ rev: 5‘-CGGGCACACACATCACAAAG-3‘
C5a receptor 1 (C5aR1)	for: 5‘-CCAGGACATGGACCCCATAG-3‘ rev: 5‘-ATGCCATCCGCAGGTATGTT-3‘
Fibroblast growth factor 23 (FGF23)	for: 5’-CAGGAGCCATGACTCGAAGG-3’ rev: 5’-CTGGGCTGAAGTGAAGCGAT-3‘
Glucose transporter 4 (GLUT4)	for: 5‘-TTATTGCAGCGCCTGAGTCT-3‘ rev: 5‘-GGGTTCCCCATCGTCAGAG-3‘)
Glutaraldehyde-phosphate dehydrogenase (GAPDH)	for: 5‘-CTTCAACAGCAACTCCCACTCTTCC-3‘ rev: 5‘-GGTGGTCCAGGGTTTCTTACTCC-3‘
Heart fatty acid binding protein (HFABP)	for: 5‘-TGACCGGAAGGTCAAGTCAC-3‘ rev: 5‘-TTAGTGTTGTCTCCTGCCCG-3
Interleukin-1β (IL-1β)	for: 5‘-GCCACCTTTTGACAGTGATGAG-3‘ rev: 5‘-TGACAGCCCAGGTCAAAGGTT-3‘
NLR family pyrin domain containing 3 (NLRP3)	for: 5‘-GCTGCTCAGCTCTGACCTCT-3‘ rev: 5‘-AGGTGAGGCTGCAGTTGTCT-3’
Sarcoplasmic/endoplasmic reticulum ATPase (SERCA)	for: 5‘-TACCTGGAACAACCCGCAAT-3‘ rev: 5‘-CTAACAACGCACATGCACGC-3‘
Toll-like receptor 2 (TLR2)	for: 5‘-GAAACCTCAGACAAAGCGTCA-3‘ rev: 5‘-ACAGCGTTTGCTGAAGAGGA-3‘
Toll-like receptor 4 (TLR4)	for: 5‘-GGACTCTGATCATGGCACTGT-3‘ rev: 5‘-GGAACTACCTCTATGCAGGGAT-3‘
Toll-like receptor 9 (TLR9)	for: 5‘-GAGAGACCCTGGTGTGGAAC-3‘ rev: 5‘-CCTTCGACGGAGAACCATGT-3‘
Troponin I	for: 5‘-GATGCGGCTGGGGAACC-3‘ rev: 5‘-ACTTTTTCTTGGCGTGTGGC-3‘
Tumor necrosis factor (TNF)	for: 5‘-GAGAGACCCTGGTGTGGAAC-3‘ rev: 5‘-CCTTCGACGGAGAACCATGT-3‘

Fibroblast growth factor 23 (FGF23) (5’-CAGGAGCCATGACTCGAAGG-3’; 5’-CTGGGCTGAAGTGAAGCGAT-3‘).

### Statistical analysis

Data were analyzed by using the GraphPad Prism 9.0 software (GraphPad Software, Inc., San Diego, CA, USA). The data were analyzed for normal distribution by using the Shapiro-Wilk test. Unless otherwise indicated, the data were distributed normally. In case of two groups (IHC and IF experiments), data were then analyzed by non-paired student t-test. In case of three or more groups (multiplex and RT-qPCR experiments), data were then analyzed by 2-way Analysis of Variance (ANOVA), followed by Sidak’s multiple comparison test. All values are expressed as mean ± SEM. p ≤ 0.05 was considered as statistically significant. Furthermore, linear regression analysis was performed between the young and the middle-aged animals at the respective time points. The goodness of fit was indicated as R^2^.

## Results

### Systemic inflammation

#### IL-6, KC and IFNγ

**Figure 1:**
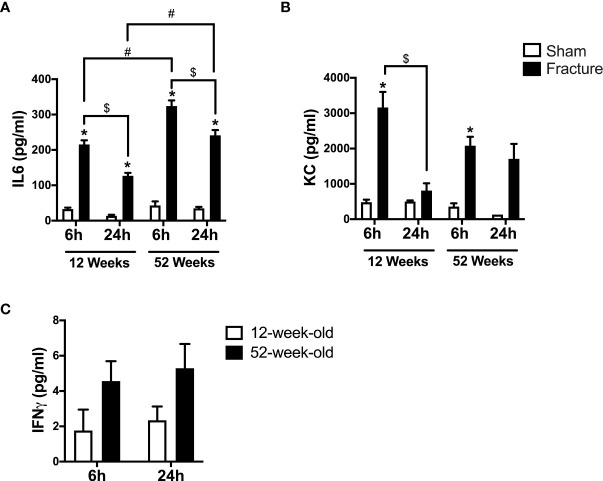
Systemic inflammation 6 and 24 h after proximal femur fracture in young and middle-aged mice. Young (12-week-old) and middle-aged (52-week-old) female mice received either sham treatment (white bars) or experimental proximal femur fracture (black bars). Blood plasma was analyzed 6 and 24 h following sham treatment or proximal femur fracture. Systemic levels of interleukin-6 (IL-6) in pg/ml **(A)**, keratinocyte chemoattractant (KC) in pg/ml **(B)** and interferon gamma (IFNγ) in pg/ml **(C)**. Data are presented as mean ± SEM. p ≤ 0.05 was considered as statistically significant. *p ≤ 0.05 sham vs. fracture. $p ≤ 0.05 6 vs. 24 h after fracture within one age group. #p ≤ 0.05 6 vs. 6h and 24 vs. 24 h between two age groups.

First, we analyzed the systemic levels of specific pro-inflammatory mediators in young and middle-aged mice 6 and 24 h after experimental proximal femur fracture or sham treatment. After 6 and 24 h, the systemic levels of interleukin 6 (IL-6) were significantly elevated in the fractured young as well as in the middle-aged mice, compared to their respective sham control groups (**Figure 1A**). Furthermore, the systemic IL-6 levels dropped 24 h after fracture compared to 6 h in the respective age groups (**Figure 1A**). When comparing both age groups, the 52-week-old mice showed significant higher IL-6 levels 6 and 24 h following fracture, compared to the 12-week-old mice (**Figure 1A**). Moreover, the middle-aged mice showed enhanced systemic IL-6 levels and a positive linear regression of systemic IL-6 levels 6 h (R^2^ = 0.76) and 24 h (R^2^ = 0.85) following hip fracture (**Supplemental Figure 2A**). Besides systemic IL-6, the systemic levels of keratinocyte chemoattractant (KC) were significantly elevated 6 h after fracture in both age groups, compared to their respective controls (**Figure 1B**). Furthermore, the systemic KC levels dropped significantly 24 h after fracture compared to 6 h in the young mice, but not in middle-aged animals (**Figure 1B**). However, in the middle-aged group the systemic KC levels also generally decreased 24 h following fracture compared to 6 h (**Figure 1B**). Moreover, the systemic levels of interferon gamma (IFNγ) were elevated in the 52-week-old animals 6 and 24 h following fracture compared to the 12-week-old animals (**Figure 1C**). Therefore, the mice showed increased systemic inflammation 6 and 24 h after proximal femur fracture, which seemed to be enhanced in the middle-aged group.

### Cardiac inflammation

#### HMGB1 and TLR signaling

**Figure 2:**
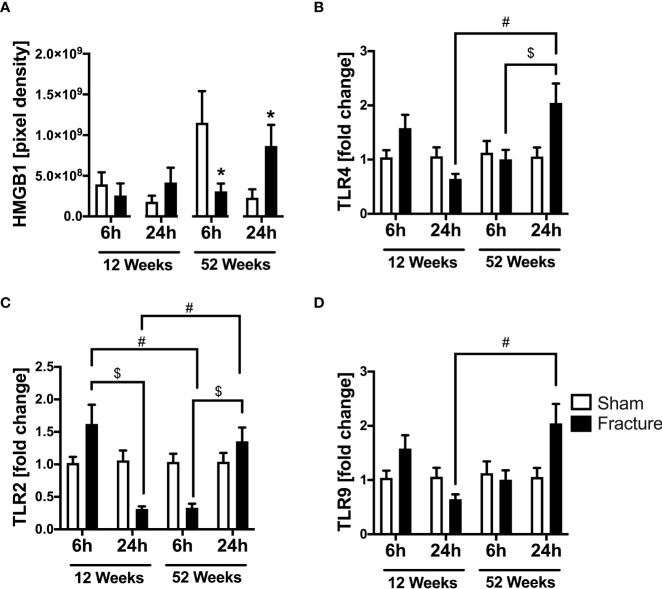
Toll-like receptor mediated cardiac inflammation 6 and 24 h after proximal femur fracture in young and middle-aged mice. Young (12-week-old) and middle-aged (52-week-old) female mice received either sham treatment (white bars) or experimental proximal femur fracture (black bars). Left ventricular cardiac tissue was analyzed 6 and 24 h following sham treatment or proximal femur fracture. Local protein expression of high-mobility group box 1 (HMGB1) protein in pixel density **(A)**, mRNA expression of toll-like receptor 4 (TLR4) in fold change **(B)**, mRNA expression of toll-like receptor 2 (TLR2) in fold change **(C)** and mRNA expression of toll-like receptor 9 (TLR9) in fold change **(D)**. Data are presented as mean ± SEM. *p ≤ 0.05 was considered as statistically significant. p ≤ 0.05 sham vs. fracture. $p ≤ 0.05 6 vs. 24 h after fracture within one age group. #p ≤ 0.05 6 vs. 6h and 24 vs. 24 h between two age groups.

We further analyzed local cardiac inflammation. We first focused on damage-associated molecular patterns (DAMPs) and toll-like receptor (TLR) signaling. In the 52-week-old mice, the high-mobility group box 1 (HMGB1) protein expression significantly decreased 6 h following fracture but significantly increased after 24 h compared to their respective control groups (**Figure 2A**). With respect to TLR signaling, the TLR4 mRNA expression was significantly higher 24 h after fracture compared to 6 h in the middle-aged group (**Figure 2B**). When comparing both age groups, the TLR4 mRNA expression was significantly higher 24 h after fracture in the middle-aged animals compared to the young animals (**Figure 2B**). Regarding the TLR2 mRNA expression, the TLR2 mRNA expression was significantly lower 24 h after fracture compared to 6 h in the young group (**Figure 2C**). In contrast, the TLR2 mRNA expression was significantly higher 24 h following fracture compared to 6 h in the middle-aged group (**Figure 2C**). When comparing young and middle-aged mice, the TLR2 mRNA expression was significantly lower 6 h following fracture in the middle-aged mice but was significantly higher after 24 h in the middle-aged group when compared to the young animals (**Figure 2C**). The TLR9 mRNA expression was significantly higher 24 h after fracture in the middle-aged mice compared to young mice (**Figure 2D**). Additionally, the middle-aged mice showed enhanced levels and a positive linear regression of TLR2 (R^2^ = 0.70), TLR4 (R^2^= 0.69) and TLR9 (R^2^ = 0.53) mRNA expression 24 h following hip fracture (**Supplemental Figures 2B–D**). Therefore, it seems like hip fracture alters local expression of TLRs in an age-dependent manner with highest expression levels 24h after fracture in middle-aged mice.

#### Complement system

**Figure 3:**
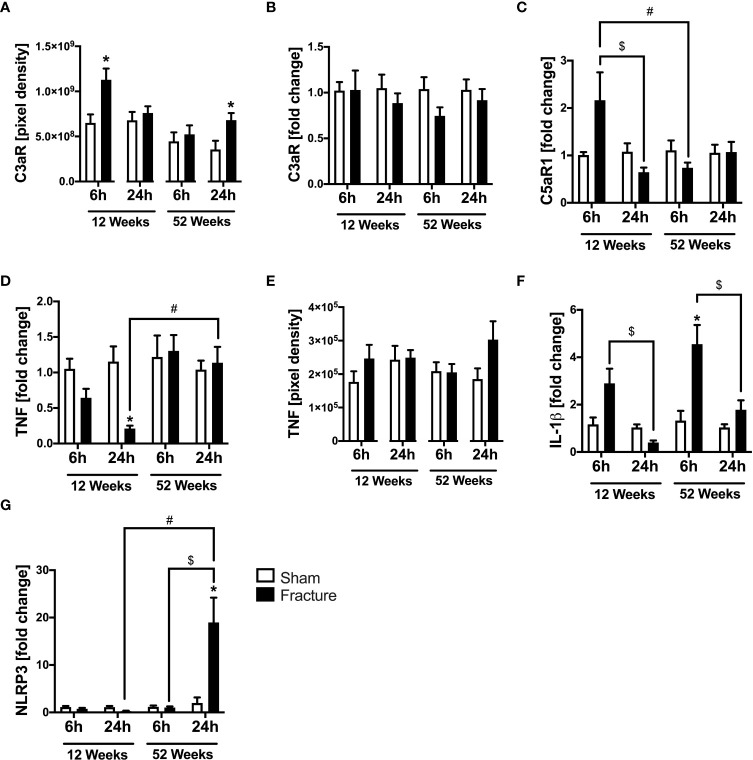
Complement system and cytokines mediated cardiac inflammation 6 and 24 h after proximal femur fracture in young and middle-aged mice. Young (12-week-old) and middle-aged (52-week-old) female mice received either sham treatment (white bars) or experimental proximal femur fracture (black bars). Left ventricular cardiac tissue was analyzed 6 and 24 h following sham treatment or proximal femur fracture. Local protein expression of C3a receptor (C3aR) in pixel density **(A)**, mRNA expression of C3a receptor (C3aR) in fold change **(B)**, mRNA expression of C5a receptor 1 (C5aR1) in fold change **(C)**, mRNA expression of tumor necrosis factor (TNF) in fold change **(D)**, protein expression of tumor necrosis factor (TNF) in pixel density **(E)**, mRNA expression of interleukin-1β (IL-1β) in fold change **(F)** and mRNA expression of NLR pyrin domain containing protein 3 (NLRP3) in fold change **(G)**. Data are presented as mean ± SEM. p ≤ 0.05 was considered as statistically significant. *p ≤ 0.05 sham vs. fracture. $p ≤ 0.05 6 vs. 24 h after fracture within one age group. #p ≤ 0.05 6 vs. 6h and 24 vs. 24 h between two age groups.

We analyzed further inflammatory pathways in ventricular tissue. With respect to the complement system, the C3a receptor (C3aR) protein expression was significantly enhanced 6 h after fracture in the young and 24 h after fracture in the middle-aged mice, compared to their respective control groups (**Figure 3A**). The C3aR mRNA expression did not differ between the respective groups (**Figure 3B**). The C5a receptor 1 (C5aR1) mRNA expression was significantly reduced 24 h after fracture compared to 6 h in the 12-week-old mice (**Figure 3C**). Also, the C5aR1 mRNA expression was significantly lower 6 h after fracture in the middle-aged mice compared to the young mice (**Figure 3C**).

#### TNF and IL-1β

Regarding pro-inflammatory cytokines, the tumor necrosis factor (TNF) mRNA expression was significantly reduced 24 h after fracture in the young mice compared to sham (**Figure 3D**). When comparing the two age groups, the TNF mRNA expression was significantly higher 24 h after fracture in the middle-aged group compared to the young group (**Figure 3D**). The myocardial TNF protein expression was slightly elevated in the 12-week-old group 6 h after fracture and in the 52-week-old group 24 h after fracture (**Figure 3E**). The interleukin-1β (IL-1β) mRNA expression was significantly elevated 6 h after fracture in the middle-aged group only compared to sham (**Figure 3F**) and dropped at 24 h compared to 6 h in the respective age groups (**Figure 3F**). Moreover, the middle-aged mice showed elevated levels and a positive linear expression of TNF (R^2^ = 0.63) and IL-1β (R^2^ = 0.53) mRNA expression 24 h following hip fracture (**Supplemental Figures 2E, G**).

#### NLRP3 inflammasome

The NLR pyrin domain containing 3 protein (NLRP3) mRNA expression significantly increased 24 h after fracture in the middle-aged group compared to sham and was also significantly higher compared to 6 h after fracture (**Figure 3G**). Likewise, the NLRP3 mRNA expression was significantly higher 24 h after fracture in the middle-aged group compared to the young animals (**Figure 3G**). These data indicate a distinct regulation of inflammatory genes in the heart after hip fracture which might be age-related. The most striking finding is the over 10-fold increase in NLRP3 gene expression 24 h after fracture in the middle-aged mice. Moreover, there were elevated levels and a positive regression of NLRP3 (R^2^ = 0.56) mRNA expression 24 h following hip fracture in the 52-week-old animals (**Supplemental Figure 2F**).

### Cardiac metabolism, oxidative system and calcium handling

#### HFABP and GLUT4

**Figure 4:**
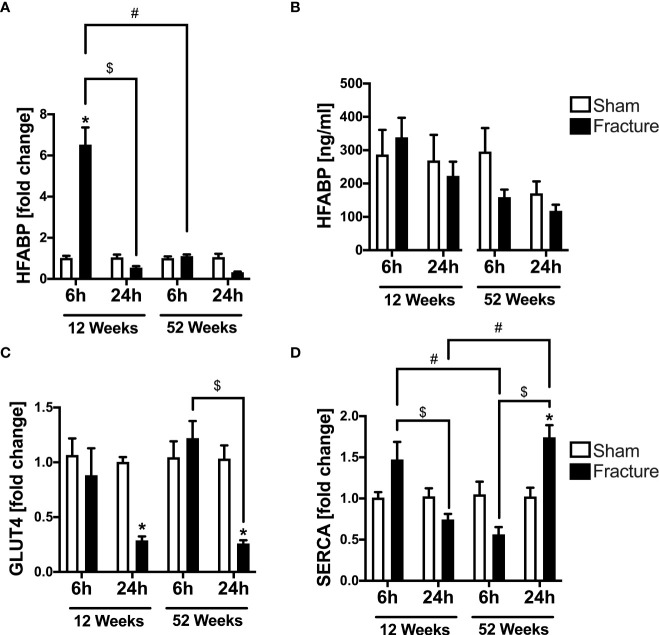
*Cardiac alterations in metabolism and calcium handling 6 and 24 h after proximal femur fracture in young and middle-aged mice.* Young (12-week-old) and middle-aged (52-week-old) female mice received either sham treatment (white bars) or experimental proximal femur fracture (black bars). Left ventricular cardiac tissue was analyzed 6 and 24 h following sham treatment or proximal femur fracture. Local mRNA expression of heart fatty acid binding protein (HFABP) in fold change **(A)**, systemic levels of heart fatty acid binding protein (HFABP) in ng/ml **(B)**, mRNA expression of glucose transporter 4 (GLUT4) in fold change **(C)** and mRNA expression of sarcoplasmic/endoplasmic reticulum ATPase (SERCA) in fold change **(D)**. Data are presented as mean ± SEM. p ≤ 0.05 was considered as statistically significant. *p ≤ 0.05 sham vs. fracture. $p ≤ 0.05 6 vs. 24 h after fracture within one age group. #p ≤ 0.05 6 vs. 6h and 24 vs. 24 h between two age groups.

We further investigated alterations in cardiac metabolism and calcium handling, since these might be caused by enhanced inflammation. With respect to cardiac fatty acid metabolism, the mRNA expression of heart fatty acid binding protein (HFABP) was significantly elevated 6 h following fracture in the young mice compared to sham (**Figure 4A**). Further, the HFABP mRNA expression was significantly lower 24 h after fracture compared to 6 h in the young mice (**Figure 4A**). When comparing the young and the middle-aged group, the HFABP mRNA expression was significantly lower 6 h following fracture in the middle-aged group (**Figure 4A**). The systemic HFABP levels slightly decreased in the middle-aged animals 6 and 24 h following hip fracture (**Figure 4B**). Regarding the cardiac glucose metabolism, the glucose transporter 4 (GLUT4) mRNA expression was significantly reduced 24 h after fracture compared to sham in both age groups (**Figure 4C**). Additionally, the GLUT4 mRNA expression was significantly lower after 24 h in the middle-aged group compared to 6 h (**Figure 4C**).

#### Serca

For myocardial calcium handling, the mRNA expression of SERCA significantly increased 24 h following fracture compared to sham in the middle-aged mice (**Figure 4D**). In the 12-week-old group, the SERCA mRNA expression was significantly lower after 24 h compared to 6 h, whereas in the 52-week-old group the SERCA mRNA expression was significantly higher 24 h following fracture compared to 6 h (**Figure 4D**). When comparing the two age groups, the SERCA mRNA expression 6 h after fracture was significantly lower in the middle-aged mice, whereas after 24 h the SERCA mRNA expression was significantly higher in the middle-aged mice (**Figure 4D**). Also, there were elevated levels and a positive regression in SERCA (R^2^ = 0.80) mRNA expression 24 h following hip fracture in the 52-week-old animals (**Supplemental Figure 2H**).

#### Nitrotyrosine, glutathione peroxidase 4 (GSH) and superoxide dismutase (SOD)

We also investigated myocardial nitrosative stress and the myocardial protein expression of anti-oxidative enzymes. With respect to nitrosative stress, the myocardial expression of nitrotyrosine was slightly elevated in the middle-aged groups 6 and 24 h following proximal femur fracture compared to sham (**Supplemental Figure 1A**). The protein expression of glutathione peroxidase 4 (GSH) in left ventricular tissue did not alter between the respective groups (**Supplemental Figure 1B**). The expression of superoxide dismutase (SOD) was slightly elevated in the middle-aged group 6 h following fracture (**Supplemental Figure 1C**).

#### Atrial natriuretic peptide (ANP), brain natriuretic peptide (BNP) and fibroblast growth factor 23 (FGF23)

We further investigated the myocardial expression of distinct myocardial markers. After 6 h, the young and middle-aged animals showed a slightly elevated myocardial mRNA expression of atrial natriuretic peptide (ANP) compared to their respective controls (**Supplemental Figure 1D**). Moreover, the brain natriuretic peptide (BNP) mRNA expression was significantly elevated 24 h following hip fracture in the young animals compared to 6 h (**Supplemental Figure 1E**). The myocardial fibroblast growth factor 23 (FGF23) mRNA expression was slightly elevated 6 and 24 h after fracture in the young and middle-aged animals compared to their respective control groups (**Supplemental Figure 1F**).

### Structural alterations

#### Heart injury score, desmin, α-actinin and troponin I

**Figure 5:**
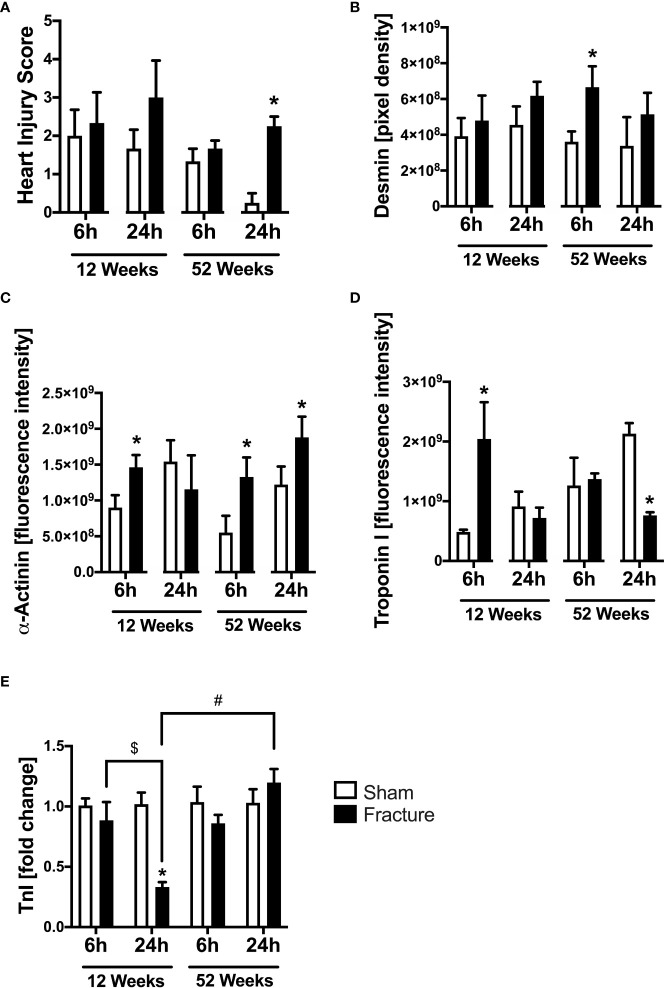
Cardiac structural alterations 6 and 24 h after proximal femur fracture in young and middle-aged mice. Young (12-week-old) and middle-aged (52-week-old) female mice received either sham treatment (white bars) or experimental proximal femur fracture (black bars). Left ventricular cardiac tissue was analyzed 6 and 24 h following sham treatment or proximal femur fracture. Myocardial Heart Injury Score **(A)**, local protein expression of Desmin in fluorescence intensity **(B)**, protein expression of α-Actinin in fluorescence intensity **(C)**, protein expression of Troponin I in fluorescence intensity **(D)** and troponin I mRNA expression in fold change **(E)**. Data are presented as mean ± SEM. p ≤ 0.05 was considered as statistically significant. *p ≤ 0.05 sham vs. fracture. $p ≤ 0.05 6 vs. 24 h after fracture within one age group. #p ≤ 0.05 6 vs. 6h and 24 vs. 24 h between two age groups.

We also investigated myocardial structural alterations following hip fracture, since these has been previously linked to enhanced cardiac inflammation. With respect to local myocardial tissue damage, the heart injury score significantly increased 24 h following fracture compared to sham in the middle-aged group only (**Figure 5A**). Regarding myocardial structure proteins, the desmin protein expression significantly increased 6 h following fracture in the middle-aged group only (**Figure 5B**). The α-actinin protein expression was significantly elevated 6 h after fracture compared to sham in the young and the middle-aged group, as well as after 24 h in the middle-aged group only (**Figure 5C**). Additionally, the troponin I protein expression significantly increased 6 h following fracture in the young mice but dropped significantly after 24 h in the middle-aged mice compared to their respective control groups (**Figure 5D**). The cardiac troponin I mRNA expression was significantly reduced in the 12-week-old group 24 h following fracture compared to sham (**Figure 5E**). Also, the myocardial troponin I mRNA expression in the young animals was significantly lower 24 h following fracture compared to 6 h (**Figure 5E**). The troponin I mRNA expression was significantly increased in the middle-aged animals 24 h following fracture compared to the young animals (**Figure 5E**). Also, there were elevated levels and a positive regression in troponin I (R^2^ = 0.83) mRNA expression 24 h following hip fracture in the middle-aged animals (**Supplemental Figure 2I**). These data indicate that local tissue damage after hip fracture is aggravated in middle-aged mice.

The results are summarized in **Table 2**.

**Table 2:** Summary results.

	Sham vs. Fx				Fx 6 h vs. 24 h		Young vs. middle-aged	
	Young 6 h Sham vs. Fx	Young 24 h Sham vs. Fx	Middle-aged 6 h Sham vs. Fx	Middle-aged 24 h Sham vs. Fx	Young 6 h vs. 24 h	Middle-aged 6 h vs. 24 h	6 h Young vs. middle-aged	24 h Young vs. middle-aged
**Systemic**								
IL-6	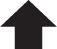	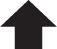	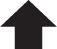	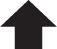	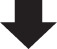	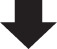	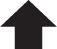	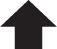
KC	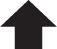		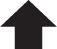		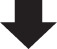			

**Local**								
HMGB1			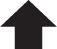	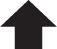				
TLR2					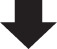	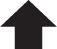	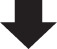	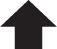
TLR4						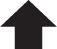		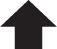
TLR9								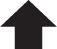
C3aR (protein)	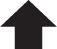			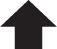				
C5aR1					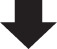		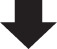	
TNF (mRNA)		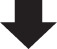						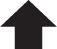
IL-1β			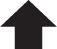		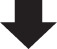	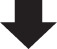		
NLRP3				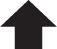		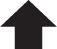		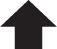
HFABP (mRNA)	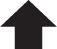				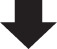		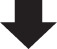	
GLUT4		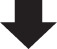		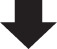		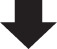		
SERCA				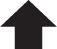	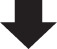	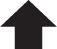	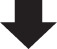	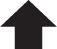
Heart Injury Score				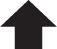				
Desmin			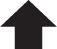					
α-actinin			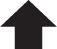	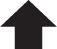				
TnI (protein)	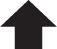			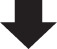				
TnI (mRNA)		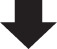			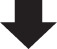			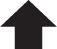
BNP					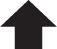			

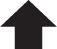
 significantly increased.

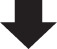
 significantly reduced

## Discussion

The aim of the present study was to investigate the effect of an experimental proximal femur fracture on the heart with respect to the development of secondary cardiac injury. Furthermore, special attention was given on the co-morbidity factor age as an independent risk factor, possibly aggravating and accelerating the development of this pathologic condition. Therefore, we investigated the systemic as well as the local cardiac inflammation 6 and 24 h following hip fracture in young (12-week-old) and middle-aged (52-week-old) mice. Additionally, local alterations in cardiac structure, metabolism and calcium handling were examined.

The development of secondary cardiac injury following long bone fracture has been recently described; it largely was linked to an immediate activation of the inflammatory response and a subsequent massive systemic release of distinct pro-inflammatory cytokines (8–10). This finding was also confirmed in the present study where mice showed increased systemic levels of IL-6 and KC following hip fracture, which seemed to be higher early after fracture at 6 h, supporting the previous findings of our group (8–10). The systemic IL-6 and KC levels were higher 6 h following fracture in both age groups and showed a slight decrease within 24 h. Both, IL-6 and KC were described as early pro-inflammatory markers following fracture (29), thus supporting the findings in the present study. Interestingly, the systemic IL-6 levels were significantly higher in the middle-aged mice at both time points following hip fracture. Enhanced systemic IL-6 levels have been described after hip fracture, particularly in elderly patients, and has been considered as an independent predictor for adverse postoperative outcomes such as complications and mortality (30). Moreover, older age of the patients was also linked to increased systemic IL-6 levels following hip fracture (31). Age-dependent differences in the pro-inflammatory response following bone fracture were demonstrated in distinct clinical and experimental studies, showing that age itself is an independent predictor for an intensified systemic inflammatory response (32). This was further confirmed in the present study, showing systemically elevated IFNγ levels in the 52-week-old mice 6 and 24 h following fracture compared to the 12-week-old animals. The phenomenon of ‘inflammaging’ was first described in the year 2000 by Franceschi and colleagues and implies an age-dependent upregulation of the inflammatory response, resulting in a low-grade chronic systemic pro-inflammatory state (33). The state of ‘inflammaging’ is characterized by enhanced systemic levels of IL-1β, IL-6 and TNF, all of which were further shown to be involved in the pathogenesis of age-associated diseases (34). This chronic pro-inflammatory state results in a vicious cycle of pathophysiological changes, tissue injury and remodeling (35). With respect to the heart, the aging-associated pro-inflammatory response was shown to be a fundamental underlying molecular mechanism for the development of myocardial dysfunction (36). More importantly, ‘inflammaging’ has been suggested as a major risk factor for the development of heart failure in geriatric patients following hip fracture and has been mostly linked to elevated systemic levels of IL-6 and TNF (32, 37). Furthermore, geriatric patients suffering from a chronic inflammatory state take longer to resolve this inflammatory response to baseline levels and show an enhanced susceptibility to infections following trauma due to a prolonged immune suppression (38). Interestingly, an enhanced systemic inflammatory response was shown in the present study in middle-aged animals, suggesting that the condition of ‘inflammaging’ not exclusively appears in geriatric patients but might be just as relevant in a younger, middle-aged population. Summarized, our data showed that experimental hip fracture induces a systemic inflammatory response, which might contribute to the development of secondary cardiac injury following hip fracture in young and middle-aged animals. Additionally, the systemic inflammatory response seemed to be aggravated in the middle-aged animals, suggesting age and the phenomenon of ‘inflammaging’ as risk factors for the development of this pathologic condition independent from pre-existing cardiac diseases.

Besides systemic inflammation, the development of secondary cardiac injury after bone fracture previously had been linked to an intensified local cardiac inflammation (8–10). To assess the extent of local cardiac inflammation, we analyzed the myocardial expression of HMGB1 as well as of TLR2, TLR4 and TLR9. In accordance with our previous findings, we detected an elevated myocardial expression of HMGB1, TLR2 and TLR4 in the middle-aged mice 24 h following hip fracture, indicating augmented myocardial inflammation after hip fracture, which might be mediated *via* DAMPs and the TLR-signaling pathways (10). Enhanced myocardial expression of HMGB1 was recently demonstrated after experimental multiple trauma in mice (27). Exposure of human and mouse cardiomyocytes (CMs) to HMGB1 *in vitro* reduced their viability and impaired their mitochondrial respiration and calcium signaling, confirming cardio-depressive effects (39). Absence of TLR2 and TLR4 improved cardiac function after ischemia/reperfusion (I/R) injury and during sepsis in mice (40, 41) and ameliorated impaired cardiac calcium handling in presence of extracellular histones (41). In the present study, the myocardial HMGB1 expression dropped significantly in the middle-aged group 6 h following fracture, which might be due to an enhanced systemic elevation of HMGB1, acting as DAMP by recruiting immune cells, thus triggering and aggravating the inflammatory response. Increased systemic HMGB1 levels following experimental hip fracture have been recently described, supporting this hypothesis (42). The significant elevation of the myocardial HMGB1 expression 24 h following hip fracture in the middle-aged group might be a compensatory effect due to enhanced systemic HMGB1 release.

In the present study, the myocardial expression of TLR2, TLR4 and TLR9 was considerably higher in the middle-aged mice compared to the young animals, suggesting that age might aggravate and accelerate myocardial inflammation following hip fracture. Age-dependent differences in TLR-mediated inflammation have already been demonstrated in previous studies. LPS treatment of murine peritoneal macrophages from aged mice showed decreased production of pro-inflammatory cytokines, which were associated with alterations in TLR4 signaling (43). Furthermore, peritoneal macrophages from middle-aged mice demonstrated an impaired ability to develop endotoxin tolerance, which was associated with age-dependent alterations in TLR2 and TLR4 and was further linked to an uncontrollable inflammation (44). Moreover, myocardial macrophages were also shown to play a key role in myocardial inflammation in the condition of ‘inflammaging’ (45). Taken together, the young and middle-aged mice showed alterations in myocardial TLR expression, suggesting a crucial role of TLR signaling in the heart following hip fracture. As for the systemic inflammation, age as an independent risk factor seemed to have a considerable effect on TLR signaling, provoking myocardial inflammation following hip fracture.

Besides TLR signaling, the development of myocardial injury following trauma has been further associated with an immediate activation of the complement system (8–10, 28, 46–48). In accordance with previous findings, the young and middle-aged mice in the present study showed an increased myocardial expression of the C3aR 6 and 24 h following hip fracture, indicating myocardial complement activation (46, 47). Moreover, the myocardial expression of the C5aR1 was significantly reduced in the middle-aged mice 6 h following hip fracture compared to the young animals. A reduced myocardial expression of the C5aR1 recently has been demonstrated by our group after experimental multiple trauma but also after isolated femur fracture and was associated further to the development of post-traumatic myocardial dysfunction and damage (9, 28).

Age-dependent differences in complement activation have been shown in previous studies. It was demonstrated that the systemic levels of the complement system protein C1q increased with aging, resulting in skeletal muscle aging, muscle fibrosis and in arterial stiffening (49–51). Moreover, the immediate activation of the complement system has been linked to an activation of the myocardial NLRP3 inflammasome, contributing to the development of septic cardiomyopathy (52). The myocardial activation of the NLRP3 inflammasome, inducing the cleavage mature IL-1β was further shown by our group after multiple trauma and isolated bone fracture, and was also considered to contribute to the development of post-traumatic myocardial damage and secondary cardiac injury (9, 10, 28). In the present study, the middle-aged mice demonstrated an increased myocardial IL-1β expression 6 h following hip fracture. Furthermore, the local expression of the NLRP3 inflammasome was strikingly elevated in the middle-aged mice after 24 h, compared to the young animals. Also, the middle-aged mice showed a significantly higher mRNA and protein expression of TNF 24 h following hip fracture compared to the young mice. Both, IL-1β and TNF were shown to act cardio-depressive on human CMs *in vitro*, contributing to the development of myocardial dysfunction and damage following trauma (9, 28). Age-dependent differences for NLRP3 inflammasome activation have been described in the literature and aged mice showed an enhanced NLRP3 inflammasome activation in alveolar macrophages, contributing to the development of pulmonary fibrosis (53). Summarized, our data showed enhanced myocardial inflammation in the young and middle-aged mice following hip fracture, which was mediated *via* the complement system and the NLRP3 inflammasome, suggesting a crucial role of these inflammatory signaling pathways. Myocardial inflammation seemed to be aggravated in the middle-aged mice, indicating age as independent risk factor, accelerating cardiac inflammation following hip fracture.

Besides myocardial inflammation, the development of secondary cardiac injuries has been further linked to local metabolic alterations following trauma. Alterations in cardiac glucose metabolism were shown during CLP sepsis and linked further to impaired cardiac function (54). Furthermore, alterations in myocardial glucose- and fatty acid transporter expression were demonstrated to be induced by inflammatory mediators (55) and might therefore be an effect of ‘inflammaging’. In the present study, the myocardial mRNA expression of HFABP was significantly reduced 6 h following hip fracture in the middle-aged mice compared to the young mice. Moreover, the myocardial GLUT4 expression was diminished 24 h after hip fracture in both young and middle-aged mice. Alterations in myocardial HFABP and GLUT4 expression have been shown by our group after multiple trauma, but also after isolated femur and tibia fracture, and were further associated with the development of post-traumatic myocardial damage (10, 55). Of course, differential oral food intake between the treatment groups cannot be excluded, which possibly influence the myocardial HFABP and GLUT4 expression. However, the alterations in cardiac glucose and fatty acid transporters following bone fracture were recently demonstrated to be induced by pro-inflammatory mediators such as HMGB1, IL-1β, IL-6 and TNF (9, 39, 55), all of which have been shown to be elevated following hip fracture in the present study. Consequently, the alterations in myocardial HFABP and GLUT4 expression in the present study might be caused by pro-inflammatory mediators. To the best of our knowledge age-dependent differences in myocardial glucose- and fatty transporter expression following bone fracture have not yet been demonstrated and might be caused by ‘inflammaging’.

Apart from metabolic alterations, the mice showed changes in their myocardial expression of the calcium handling protein SERCA. Alterations in myocardial SERCA expression have been associated with the development of septic cardiomyopathy (56) and post-traumatic myocardial damage (57), which was mediated by pro-inflammatory mediators (46, 57). In the present study, the middle-aged mice showed a significantly reduced SERCA expression 6 h following hip fracture compared to the young animals, whereas after 24 h the myocardial SERCA expression was significantly elevated in the middle-aged animals compared to the young animals. Reduced myocardial SERCA expression and activity have been described in previous studies in mice and has been linked to progressive aging of the heart (58). Furthermore, a diminished SERCA expression was shown in 24-month-old mice after femur fracture and hemorrhage, which was associated with an enhanced mortality rate of the animals following trauma (59). Moreover, alterations in myocardial SERCA expression were shown to be induced by the complement activation products C3a and C5a *in vitro* (46). Consequently, alterations in myocardial SERCA expression might also be caused by ‘inflammaging’. To summarize, the mice showed alterations in myocardial expression of glucose- and fatty acid transporters as well as of calcium handling proteins following hip fracture. As for myocardial inflammation, age seemed to be an independent risk factor aggravating the cardiac effects following hip fracture.

Cardiac structural alterations have been associated with the development of post-traumatic myocardial damage and were further linked to inflammation (57). The current study demonstrated that middle-aged mice showed an increased elevation of their heart injury score 24 h after hip fracture, indicating myocardial tissue damage. Moreover, the desmin expression was also significantly elevated in the middle-aged mice after 6 h, whereas the α-actinin expression was increased in the young and middle-aged mice 6 and 24 h following hip fracture. Alterations in myocardial desmin and α-actinin expression were described after severe multiple trauma and isolated long bone fracture, and have been further associated with the development of post-traumatic myocardial dysfunction (9, 10, 28, 57). In different recent experimental studies, age- and gender-related alterations in myocardial desmin expression were demonstrated. Female 14-week-old C57BL/6 wild type mice showed an increased myocardial desmin expression compared to 100-week-old female mice (60). Further, the phosphorylation of desmin in the heart increased with age only in male mice, whereas the troponin phosphorylation only increased in female mice with age, both leading to an impaired calcium signaling of ventricular CMs (61). In the present study, the troponin I expression was significantly elevated in the young mice after 6 h and was reduced in the middle-aged animals 24 h after fracture, indicating an age-dependent effect on myocardial troponin I expression following hip fracture. Interestingly, cardiac troponin T was shown to be expressed in skeletal muscle with age and its mRNA levels were considerably higher in older compared to younger adults (62). Additionally, increased cardiac troponin T expression in skeletal muscle was demonstrated to play a critical role in in mediating neuromuscular junction denervation in skeletal muscle (63), skeletal muscle degeneration and a decline in motor activity in old mice (64). Alterations in myocardial structure protein expression following trauma were also linked to an enhanced inflammatory response. Treatment of human CMs with a defined polytrauma cocktail induced alterations in cellular troponin I expression (57). As troponin I is an essential protein for cardiomyocyte contraction, the myocardial alterations in troponin I expression in the present study might indicate for adaption of cardiomyocyte contraction proteins, thus maintaining proper cardiac function. Taken together, this study showed alterations in the expression of important myocardial structure proteins, possibly predisposing for the development of secondary cardiac injury following hip fracture. To the best of our knowledge, these age-dependent alterations in myocardial structure following hip fracture have not been demonstrated previously. Moreover, age seemed to be an independent risk factor aggravating and accelerating myocardial damage and structural alterations, consistent contextually in the scope of ‘inflammaging’. Besides age, gender differences might additionally play an important role on the heart following hip fracture, which has to be evaluated further in future studies.

There are several limitations in the current study to consider when interpreting the data. One limitation is that relatively short observation time points of 6 and 24 h following hip fracture were studied. These observation time points were chosen to investigate the early myocardial alterations following hip fracture, which is the primary research focus of our group. Therefore, the long-term effects of hip fracture on the heart with respect to age differences were not evaluated. Nevertheless, the linear regression analysis in the present study indicated myocardial alterations mainly 24 h following hip fracture and therefore suggest also later effects on the heart, which has to be included in future studies. Also, the study from De’Ath et al. showed the development of TISCI 72 h following trauma (6), wherefore later timepoints should be considered for future studies to assess the time-dependent development of myocardial alterations following hip fracture in young and middle-aged mice. Another limitation is that the present study only evaluated young (12-week-old) and middle-aged (52-week-old) mice. The middle-aged mice correspond to approximately 60 years of human age. However, the incidence of hip fractures is more commonly seen in elderly individuals greater than 75 years. Moreover, women over the age of 65 years have been shown to be more affected by hip fractures than men due to the onset of osteoporosis (65). To address this impact, elderly animals should be studied in future. Additionally, gender differences should be considered in follow-up studies since age, in combination with gender, seems to affect the clinical outcome of hip fracture patients. Also, osteoporosis should also be evaluated as a co-morbidity factor, since it is known that 52-week-old mice have age-related osteoporosis (66). Moreover, to properly analyze the extent of secondary cardiac injury following hip fracture, cardiac functional analysis by echocardiography or cardio MRI are mandatory for future studies. Finally, the model of proximal femur fracture used in this study possibly induces less soft tissue trauma than a closed fracture and could induce less of an inflammatory response than that seen clinically. Additionally, the experimental fracture was stabilized immediately following fracture, whereas hip fractures typically are stabilized within 24 h. Therefore, the hip fracture model in this study is not completely applicable to the human clinical condition.

## Conclusion

In conclusion, young and middle-aged mice showed alterations in myocardial structure, glucose- and fatty acid transport as well as in calcium homeostasis 6 and 24 h following experimental hip fracture, which might predispose for the development of secondary cardiac injury. Also, the young and middle-aged mice demonstrated an elevated systemic and local cardiac inflammatory responses following hip fracture. Interestingly, the myocardial alterations as well as the systemic and local inflammation seemed to be aggravated in the middle-aged mice, indicating age and the age-associated phenomenon of ‘inflammaging’ as an independent risk factor aggravating and accelerating cardiac alterations and therefore possibly also the development of secondary cardiac injury following hip fracture.

In the present study, we showed for the first time cardiac alterations following hip fracture in young and middle-aged animals in absence of pre-existing cardiac diseases. More importantly, the middle-aged animals demonstrated aggravated systemic and local cardiac inflammation. To the best of our knowledge, we showed for the first time that the condition of ‘inflammaging’ also occur in middle-aged animals, suggesting even middle age as independent risk factor for cardiac alterations following hip fracture.

## Data availability statement

The original contributions presented in the study are included in the article/**Supplementary Material**. Further inquiries can be directed to the corresponding authors.

## Ethics statement

The animal study was reviewed and approved by IACUC UCSF AN143402-03B.

## Author contributions

IL, BW, AO, CL, MH-L, and MK, conducting experiments. MK, MH-L, BW, JP, RM, and TM, substantial contributions to research design. MK, RM, JP, and TM, interpretation of data. IL, drafting and writing the paper. All authors, revising the paper critically and approve the final version of this paper.

## Funding

This study was supported by the Hertha-Nathorff program (travel grant to MH-L and MK), the DAAD (travel grant to BW), and the Orthopaedic Trauma Institute at UCSF. This work was conducted in the framework of the CRC1149 funded by the Deutsche Forschungsgemeinschaft (DFG, German Research Foundation) – Project number 251293561.

## Conflict of interest

The authors declare that the research was conducted in the absence of any commercial or financial relationships that could be construed as a potential conflict of interest.

## Publisher’s note

All claims expressed in this article are solely those of the authors and do not necessarily represent those of their affiliated organizations, or those of the publisher, the editors and the reviewers. Any product that may be evaluated in this article, or claim that may be made by its manufacturer, is not guaranteed or endorsed by the publisher.

## Supplementary material

Supplementary Figure 1 Cardiac nitrosative and oxidative stress and myocardial markers 6 and 24 h after proximal femur fracture in young and middle-aged mice. Young (12-week-old) and middle-aged (52-week-old) female mice received either sham treatment (white bars) or experimental proximal femur fracture (black bars). Left ventricular cardiac tissue was analyzed 6 and 24 h following sham treatment or proximal femur fracture. Myocardial expression of nitrotyrosine in pixel density **(A)**, protein expression of glutathione peroxidase (GSH) in pixel density **(B)**, protein expression of superoxide dismutase (SOD) in pixel density **(C)**, mRNA expression of atrial natriuretic peptide (ANP) in fold change **(D)**, mRNA expression of brain natriuretic peptide (BNP) in fold change **(E)** and fibroblast growth factor 23 (FGF23) in fold change **(F)**. Data are presented as mean ± SEM. p ≤ 0.05 was considered as statistically significant. *p ≤ 0.05 sham vs. fracture. $p ≤ 0.05 6 vs. 24 h after fracture within one age group. #p ≤ 0.05 6 vs. 6h and 24 vs. 24 h between two age groups.

Supplementary Figure 2 Regression analysis 6 and 24 h after proximal femur fracture in young and middle-aged mice. Young (12-week-old) and middle-aged (52-week-old) female mice received experimental proximal femur fracture. Regression analysis was performed either after 6 h (blue dots) or after 24 h (green squares) following experimental proximal femur fracture. Linear regression analysis of systemic interleukin-6 (IL-6) levels in pg/ml **(A)**, of cardiac mRNA expression of toll-like receptor 2 (TLR) **(B)**, toll-like receptor 4 (TLR4) **(C)**, toll-like receptor 9 (TLR9) **(D)**, tumor necrosis factor (TNF) **(E)**, NLR pyrin domain containing protein 3 (NLRP3) **(F)**, interleukin-1β (IL-1β) **(G)**, sarcoplasmic/endoplasmic reticulum ATPase (SERCA) **(H)** and troponin I **(I)** in fold change. Data are presented as individual values. Goodness of fit is indicated as R^2^.

Supplementary Figure 3 Representative images of C3aR and HMGB1 IHC staining of left ventricular tissue from young and middle-aged mice 6 and 24 h following proximal femur fracture. Young (12-week-old) and middle-aged (52-week-old) female mice received experimental proximal femur fracture. Left ventricular cardiac tissue was analyzed 6 and 24 h following sham treatment or proximal femur fracture. Representative images of complement C3a receptor (C3aR) **(A)** and high mobility group box one (HMGB1) protein **(B)**.

Supplementary Figure 4 Representative images of TNF IHC and H.E. staining of left ventricular tissue from young and middle-aged mice 6 and 24 h following proximal femur fracture. Young (12-week-old) and middle-aged (52-week-old) female mice received experimental proximal femur fracture. Left ventricular cardiac tissue was analyzed 6 and 24 h following sham treatment or proximal femur fracture. Representative images of tumor necrosis factor (TNF) **(A)** and hematoxylin & eosin (H.E.) staining **(B)**.

Supplementary Figure 5 Representative images of α-actinin and desmin IF staining of left ventricular tissue from young and middle-aged mice 6 and 24 h following proximal femur fracture. Young (12-week-old) and middle-aged (52-week-old) female mice received experimental proximal femur fracture. Left ventricular cardiac tissue was analyzed 6 and 24 h following sham treatment or proximal femur fracture. Representative images of α-actinin **(A)** and desmin protein **(B)**.

Supplementary Figure 6 Representative images of troponin I IF staining of left ventricular tissue from young and middle-aged mice 6 and 24 h following proximal femur fracture. Young (12-week-old) and middle-aged (52-week-old) female mice received experimental proximal femur fracture. Left ventricular cardiac tissue was analyzed 6 and 24 h following sham treatment or proximal femur fracture. Representative images of troponin I **(A)**.
