# Supplementary figures and images for: Cardiac alterations following experimental hip fracture - inflammaging as independent risk factor
Source: Front Immunol. 2022 Sep 5;13:895888. doi: 10.3389/fimmu.2022.895888 (PMC9484325; doi:10.3389/fimmu.2022.895888)

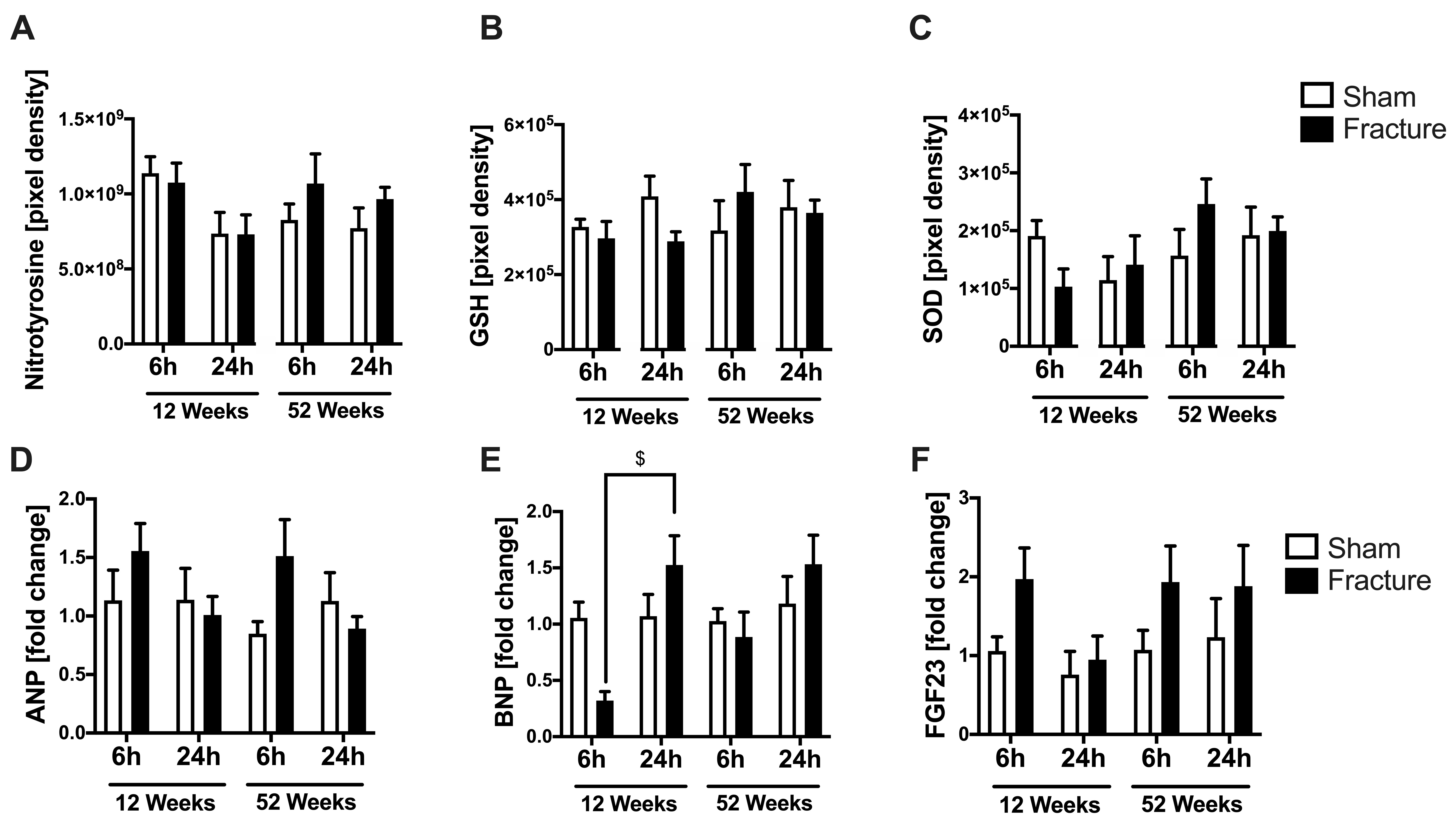

Supplement: Supplementary Figure 1 — Cardiac nitrosative and oxidative stress and myocardial markers 6 and 24 h after proximal femur fracture in young and middle-aged mice. Young (12-week-old) and middle-aged (52-week-old) female mice received either sham treatment (white bars) or experimental proximal femur fracture (black bars). Left ventricular cardiac tissue was analyzed 6 and 24 h following sham treatment or proximal femur fracture. Myocardial expression of nitrotyrosine in pixel density (A), protein expression of glutathione peroxidase (GSH) in pixel density (B), protein expression of superoxide dismutase (SOD) in pixel density (C), mRNA expression of atrial natriuretic peptide (ANP) in fold change (D), mRNA expression of brain natriuretic peptide (BNP) in fold change (E) and fibroblast growth factor 23 (FGF23) in fold change (F). Data are presented as mean ± SEM. p ≤ 0.05 was considered as statistically significant. *p ≤ 0.05 sham vs. fracture. $p ≤ 0.05 6 vs. 24 h after fracture within one age group. #p ≤ 0.05 6 vs. 6h and 24 vs. 24 h between two age groups. [file Image_1.jpeg]

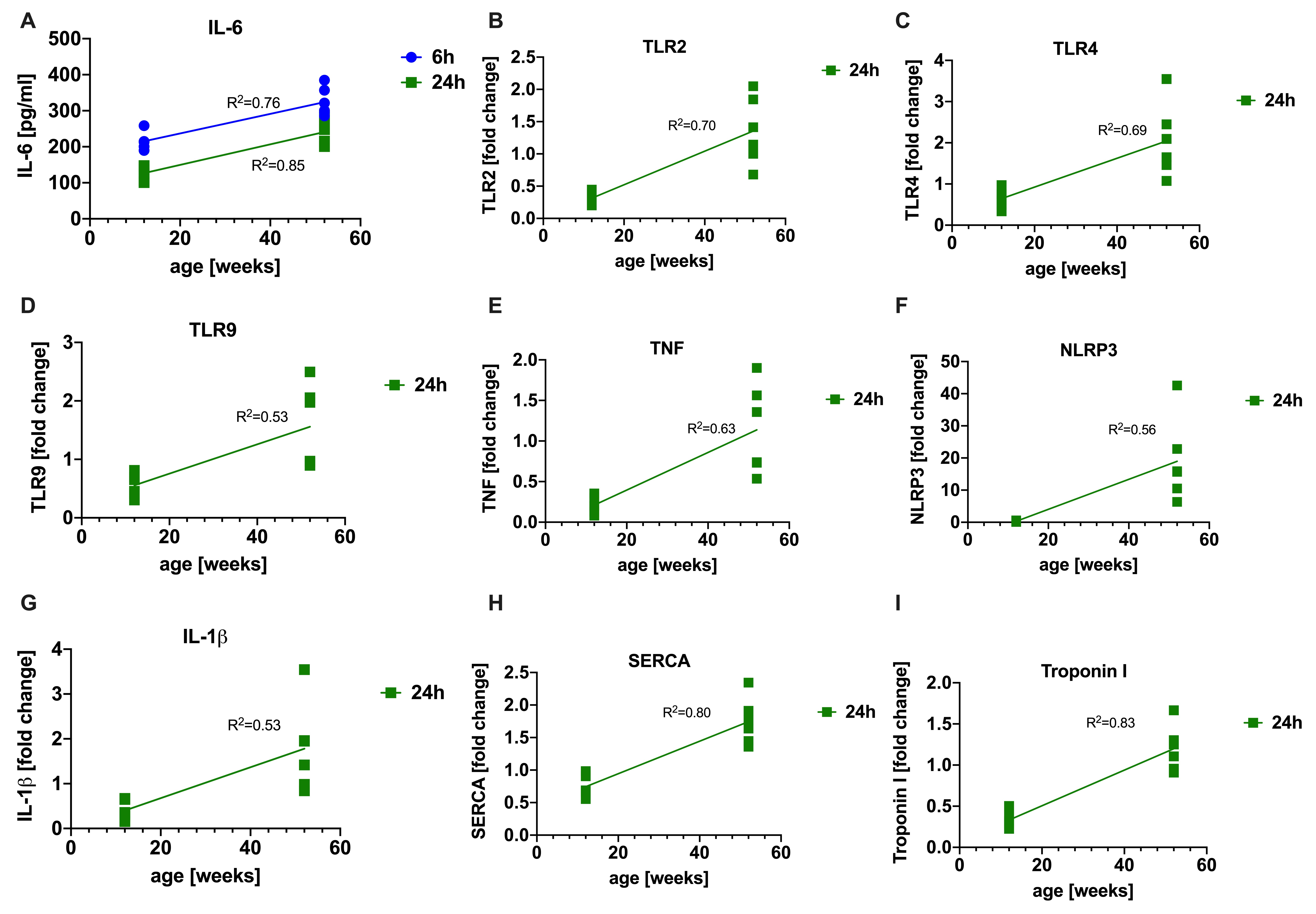

Supplement: Supplementary Figure 2 — Regression analysis 6 and 24 h after proximal femur fracture in young and middle-aged mice. Young (12-week-old) and middle-aged (52-week-old) female mice received experimental proximal femur fracture. Regression analysis was performed either after 6 h (blue dots) or after 24 h (green squares) following experimental proximal femur fracture. Linear regression analysis of systemic interleukin-6 (IL-6) levels in pg/ml (A), of cardiac mRNA expression of toll-like receptor 2 (TLR) (B), toll-like receptor 4 (TLR4) (C), toll-like receptor 9 (TLR9) (D), tumor necrosis factor (TNF) (E), NLR pyrin domain containing protein 3 (NLRP3) (F), interleukin-1β (IL-1β) (G), sarcoplasmic/endoplasmic reticulum ATPase (SERCA) (H) and troponin I (I) in fold change. Data are presented as individual values. Goodness of fit is indicated as R2. [file Image_2.jpeg]

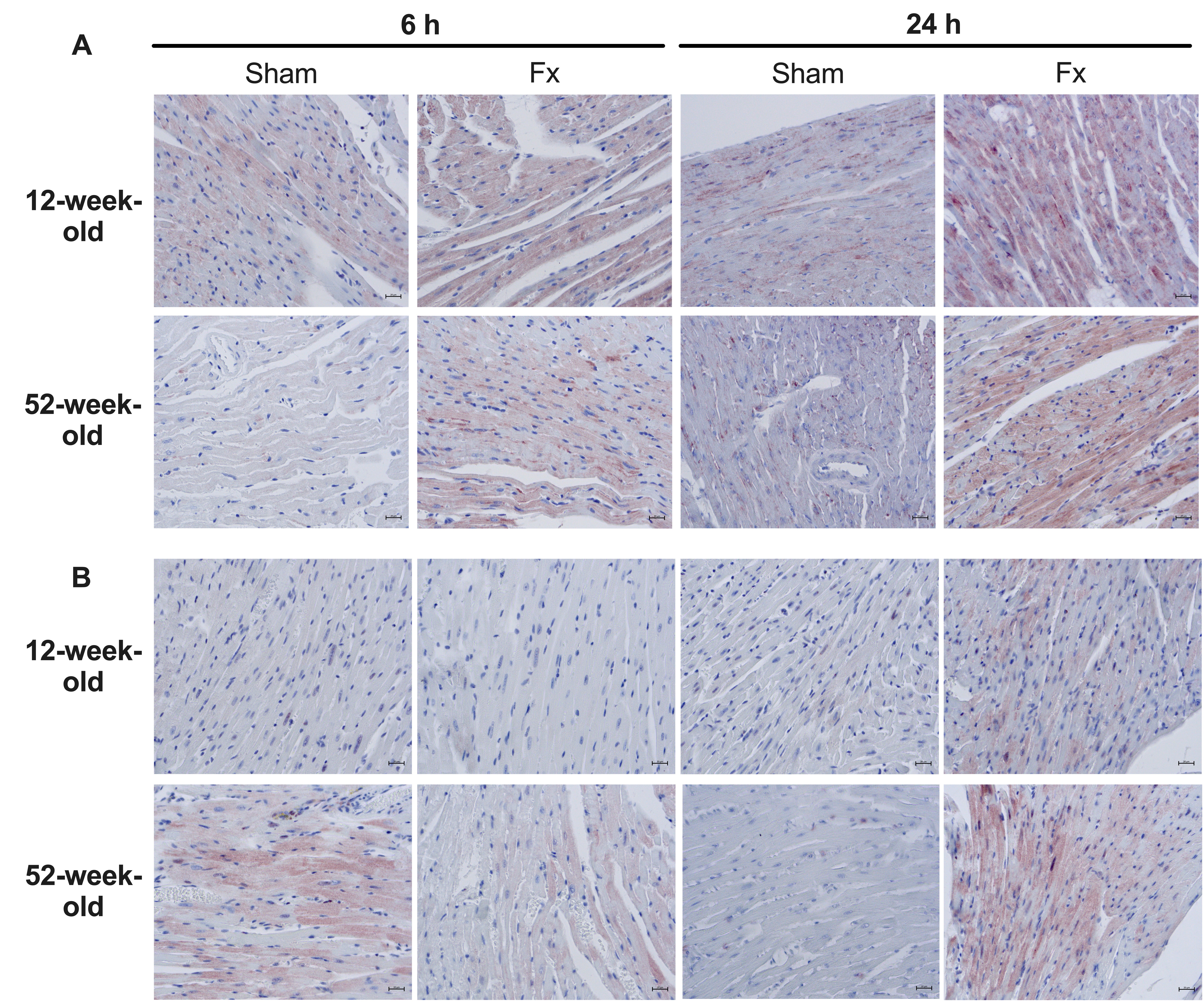

Supplement: Supplementary Figure 3 — Representative images of C3aR and HMGB1 IHC staining of left ventricular tissue from young and middle-aged mice 6 and 24 h following proximal femur fracture. Young (12-week-old) and middle-aged (52-week-old) female mice received experimental proximal femur fracture. Left ventricular cardiac tissue was analyzed 6 and 24 h following sham treatment or proximal femur fracture. Representative images of complement C3a receptor (C3aR) (A) and high mobility group box one (HMGB1) protein (B). [file Image_3.jpeg]

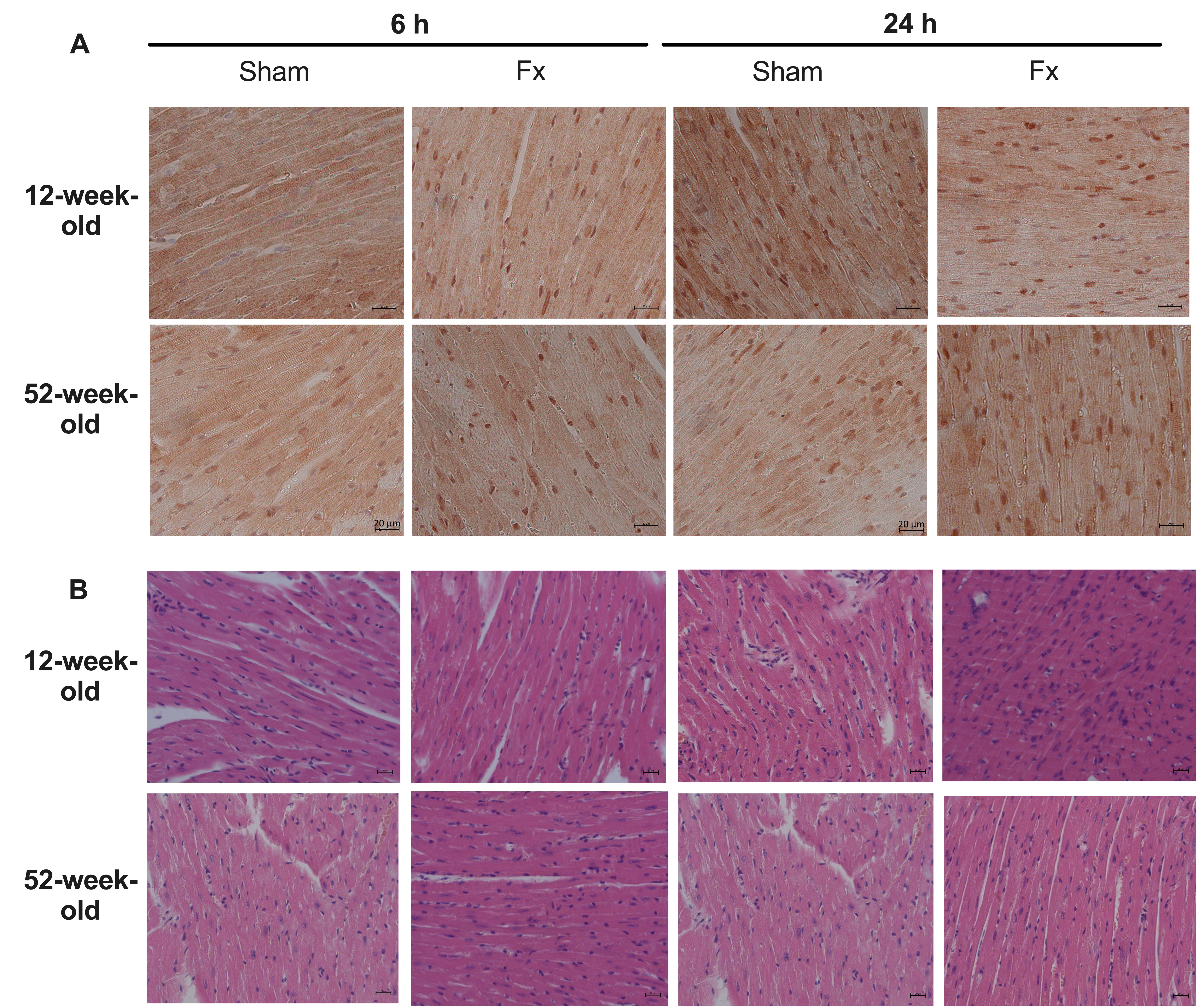

Supplement: Supplementary Figure 4 — Representative images of TNF IHC and H.E. staining of left ventricular tissue from young and middle-aged mice 6 and 24 h following proximal femur fracture. Young (12-week-old) and middle-aged (52-week-old) female mice received experimental proximal femur fracture. Left ventricular cardiac tissue was analyzed 6 and 24 h following sham treatment or proximal femur fracture. Representative images of tumor necrosis factor (TNF) (A) and hematoxylin & eosin (H.E.) staining (B). [file Image_4.jpeg]

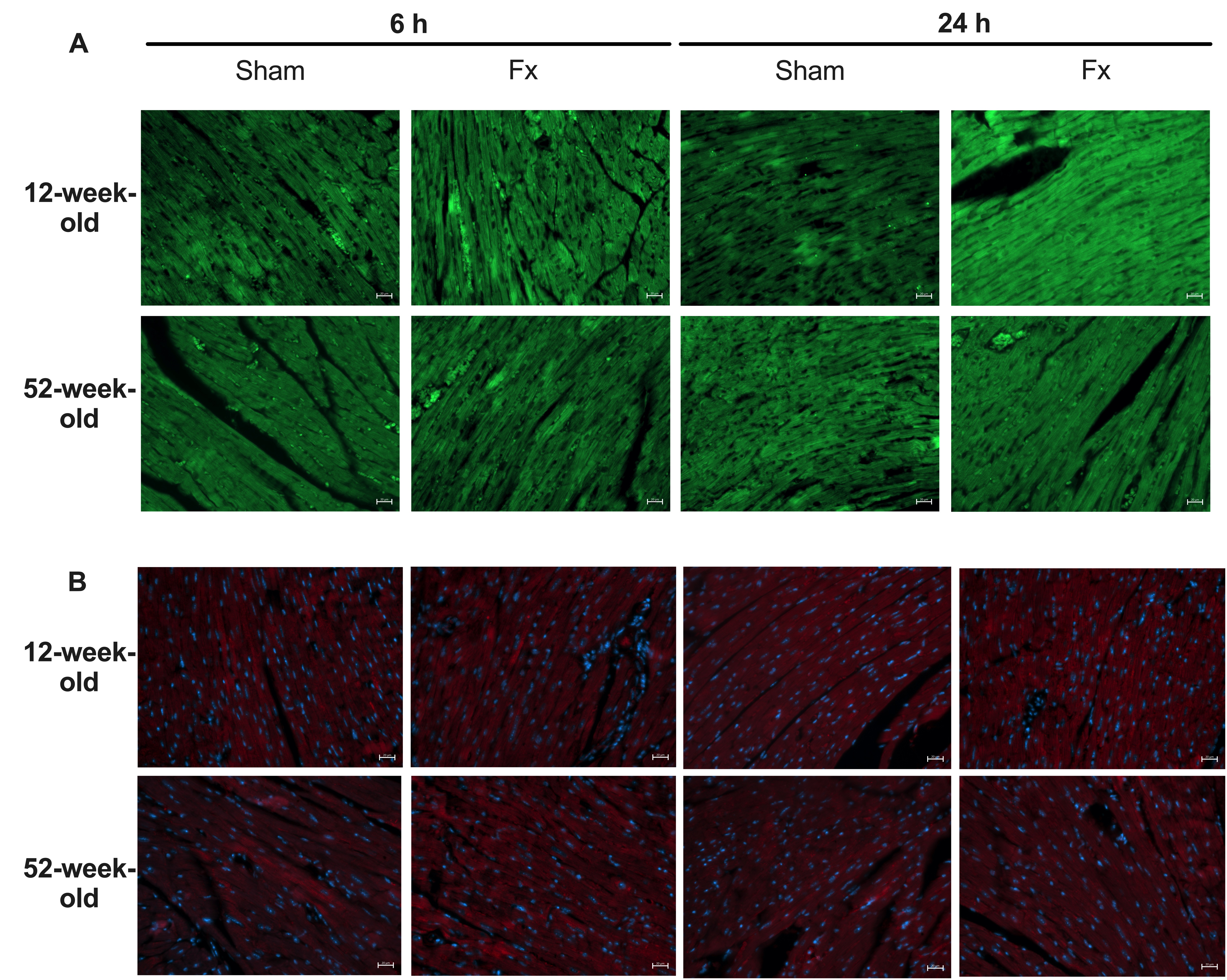

Supplement: Supplementary Figure 5 — Representative images of α-actinin and desmin IF staining of left ventricular tissue from young and middle-aged mice 6 and 24 h following proximal femur fracture. Young (12-week-old) and middle-aged (52-week-old) female mice received experimental proximal femur fracture. Left ventricular cardiac tissue was analyzed 6 and 24 h following sham treatment or proximal femur fracture. Representative images of α-actinin (A) and desmin protein (B). [file Image_5.jpeg]

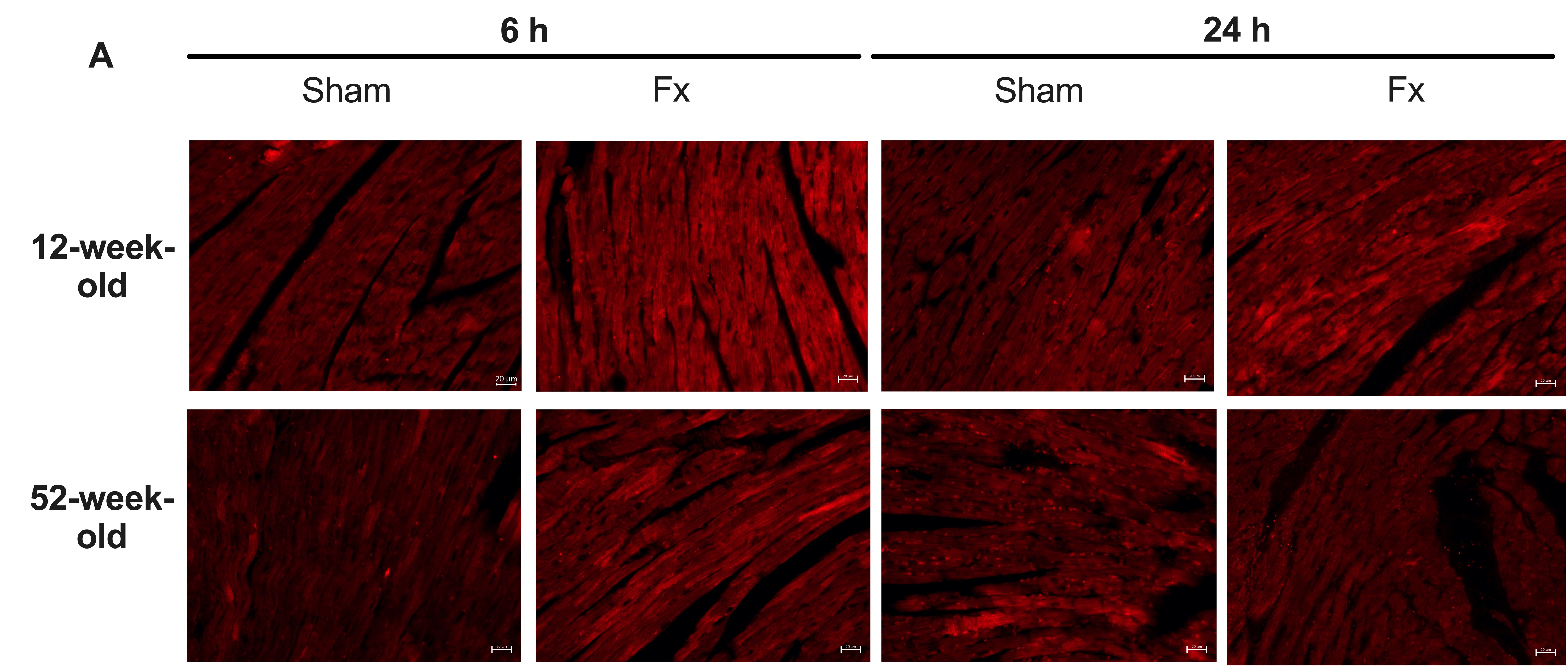

Supplement: Supplementary Figure 6 — Representative images of troponin I IF staining of left ventricular tissue from young and middle-aged mice 6 and 24 h following proximal femur fracture. Young (12-week-old) and middle-aged (52-week-old) female mice received experimental proximal femur fracture. Left ventricular cardiac tissue was analyzed 6 and 24 h following sham treatment or proximal femur fracture. Representative images of troponin I (A). [file Image_6.jpeg]
